# Activation of G-protein-gated inwardly rectifying potassium (Kir3/GirK) channels rescues hippocampal functions in a mouse model of early amyloid-*β* pathology

**DOI:** 10.1038/s41598-017-15306-8

**Published:** 2017-11-07

**Authors:** Irene Sánchez-Rodríguez, Sara Temprano-Carazo, Alberto Nájera, Souhail Djebari, Javier Yajeya, Agnès Gruart, José M. Delgado-García, Lydia Jiménez-Díaz, Juan D. Navarro-López

**Affiliations:** 1 University of Castilla-La Mancha, NeuroPhysiology & Behavior Laboratory, Centro Regional de Investigaciones Biomédicas, School of Medicine of Ciudad Real, Ciudad Real, Spain; 2 University of Salamanca, Instituto de Neurociencias de Castilla y León, Salamanca, Spain; 3 Pablo de Olavide University, Division of Neurosciences, Seville, Spain

**Keywords:** Hippocampus, Inhibition-excitation balance, Neurophysiology

## Abstract

The hippocampus plays a critical role in learning and memory. Its correct performance relies on excitatory/inhibitory synaptic transmission balance. In early stages of Alzheimer’s disease (AD), neuronal hyperexcitability leads to network dysfunction observed in cortical regions such as the hippocampus. G-protein-gated potassium (GirK) channels induce neurons to hyperpolarize, contribute to the resting membrane potential and could compensate any excesses of excitation. Here, we have studied the relationship between GirK channels and hippocampal function in a mouse model of early AD pathology. Intracerebroventricular injections of amyloid-*β* (A*β*_1-42_) peptide—which have a causal role in AD pathogenesis—were performed to evaluate CA3–CA1 hippocampal synapse functionality in behaving mice. A*β* increased the excitability of the CA3–CA1 synapse, impaired long-term potentiation (LTP) and hippocampal oscillatory activity, and induced deficits in novel object recognition (NOR) tests. Injection of ML297 alone, a selective GirK activator, was also translated in LTP and NOR deficits. However, increasing GirK activity rescued all hippocampal deficits induced by A*β* due to the restoration of excitability values in the CA3–CA1 synapse. Our results show a synaptic mechanism, through GirK channel modulation, for the prevention of the hyperexcitability that causally contributes to synaptic, network, and cognitive deficits found in early AD pathogenesis.

## Introduction

The leading cause of dementia, Alzheimer’s disease (AD), is characterized by a progressive neurodegeneration associated to a loss of memory and impairment of other cognitive functions. The neuropathological hallmarks of AD are extracellular senile plaques, consisting predominantly of amyloid-*β* (A*β*) deposits, and intracellular neurofibrillary tangles, consisting of hyperphosphorylated *tau* protein^1^. In AD, the hippocampal system, which is well known to play a critical role in learning and memory processes, is affected early^2,3^. Its correct performance relies on excitatory/inhibitory synaptic transmission balance, which has been shown to be a pivotal target of A*β*^4^. Since, in early stages of AD, hippocampal neuronal circuits become predominantly hyperactive instead of hypoactive^5^, it has been suggested that restoring the balance lost in early AD *via* increasing inhibitory signals may prevent neuronal dysfunction and cognitive impairments associated to this dementia^6–8^.

The G-protein-gated inwardly rectifying potassium (Kir3/GirK) channel is the effector of many neurotransmitters, such as GABA, dopamine, serotonin or adenosine among others (for a review^9^) and can also be constitutively active^10^. Its activation controls neuronal excitability by neuronal hyperpolarization^11^ and contributes to resting conductances^10,12^. They are homo- and heterotetramers formed by GIRK1−4 subunits^13^. In the brain, GirK channels are heterotetramers of GIRK1/GIRK2, GIRK1/GIRK3, or GIRK2/GIRK3^9^, although there is general agreement that GIRK1⁄GIRK2 heteromultimers are the neural prototypical GirK channel^11,14,15^. GirK channel dysfunction has been linked to pathological states related with impairments in the excitatory/inhibitory neuronal activity balance such as epilepsy^16^ and Down syndrome^17^, both widely related with AD^18^. In fact, it has been described *in vitro* an unrecognized aberrant function of the GirK channel in AD-related synaptic pathophysiology^18–20^ leading to increased neuronal excitability. Neuronal hyperexcitability occurs early in the pathogenesis of AD, contributing to network dysfunction both in patients and transgenic A*β*-overexpressing mice models of the disease^21–24^, and recent clinical evidence shows that reduction of hippocampal hyperactivity improves cognition in early AD^6^. Therefore, we wondered whether GirK function might be a relevant key in hippocampal AD pathology. To address this issue, we have investigated, in alert behaving mice, the impact of local A*β* injections on both hippocampal synaptic and oscillatory properties, and hippocampal-dependent cognitive functions. Our data suggest that GirK channels are necessary for normal hippocampal functionality. Interestingly, we found that increasing GirK activity restores hippocampal synaptic plasticity and network activity, and overcomes memory deficits induced by A*β*, supporting the contention that manipulations focused on the re-establishment of network excitation/inhibition balance by preventing network hyperexcitability would provide new therapeutic approaches in the pathogenesis of AD^5,18,25^. Together, our results provide a potential synaptic mechanism through GirK channels to oppose A*β* synaptic, network and cognitive hippocampal dysfunctions.

## Results

**Figure 1:**
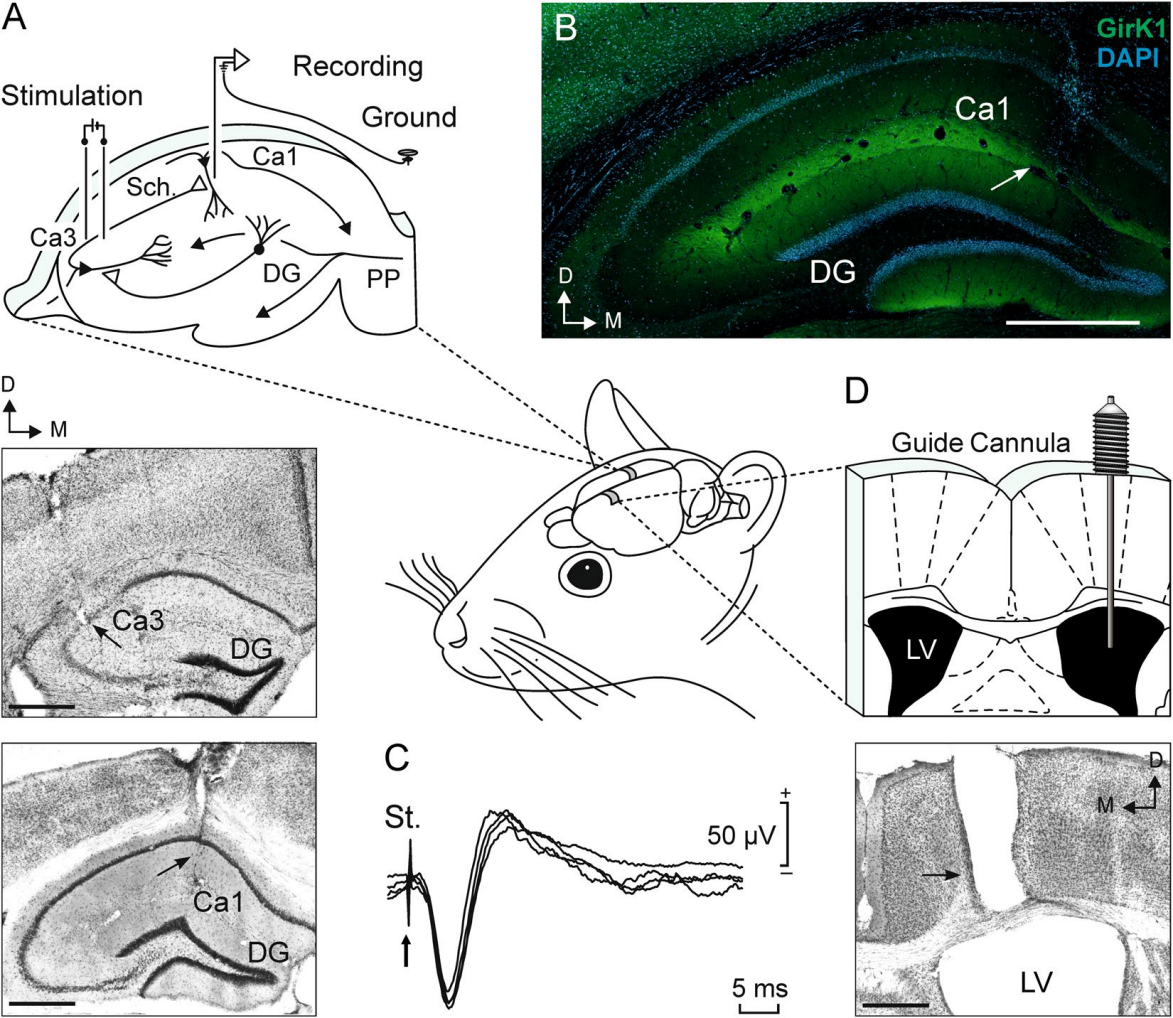
Experimental design. (**A**) The diagram illustrates how mice were prepared for chronic recording of fEPSPs evoked at the hippocampal CA3−CA1 synapse. Bipolar stimulation electrodes were surgically implanted on the right Schaffer collaterals [2 mm lateral and 1.5 mm posterior to bregma and 1.3 mm from the brain surface^75^], while a bipolar recording electrode was aimed at ipsilateral CA1 area [1.2 mm lateral and 2.2 mm posterior to bregma and 1.3 mm from the brain surface]. A bare silver wire affixed to the bone served as ground. Photomicrographs illustrate the histological verification of electrodes position. Black arrows indicate the location of the stimulating (upper photomicrograph) and recording (bottom photomicrograph) electrodes. (**B**) Confocal fluorescence photomicrograph showing the distribution of GirK1 subunit (green labelling), with intense immunolabeling in the *stratum lacunosum-moleculare* and distal parts of the *stratum radiatum* and the *stratum moleculare*^87^, and DAPI stained cells (blue labelling) in the hippocampus. Note that the recording electrode, indicated by white arrow, reaches the hippocampal layer where immunoreactivity for GirK1 is stronger. (**C**) fEPSP profile evoked by single pulses collected from a representative animal at intermediate stimulus intensities. (**D**) The diagram illustrates how mice were prepared for drug administration. A stainless steel guide cannula was implanted contralaterally to both electrodes, on the left ventricle [1 mm lateral and 0.5 mm posterior to bregma and 1.8 mm from the brain surface]. The photomicrograph serve as histological verification of cannula position (black arrow). Scale bars, 500 *μ*m. LV, Lateral Ventricle; DG, Dentate gyrus; PP, Perforant pathway; Sch., Schaffer Collateral; St., stimulus; D, dorsal; M, medial.

As detailed in Methods, experimental mice were prepared for the chronic recording of field postsynaptic potentials (fPSPs) at CA3–CA1 synapses and intracerebroventricular (*i.c.v*.) injections (Fig. 1). Both recordings and injections were performed in freely moving animals.

### GirK channel activation prevents the increase of excitability induced by A*β* at the hippocampal CA3–CA1 synapse

**Figure 2:**
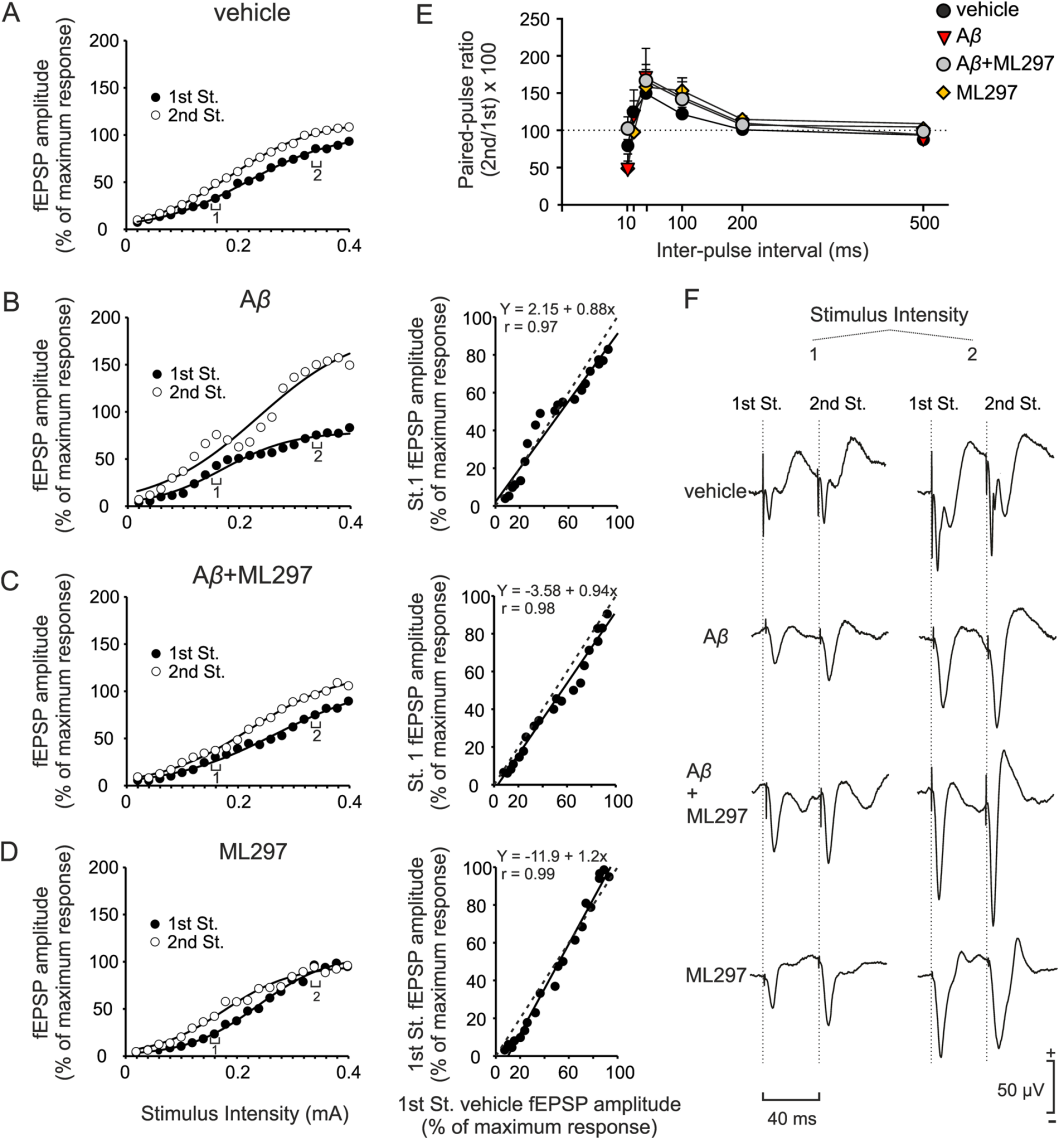
I/O and PPF curves at the CA3–CA1 synapse in *i.c.v*. injected animals. (**A**) and left panel of (**B**),(**C**) and (**D**), Relationships between the intensity (in mA) of pairs of stimuli (40 ms interstimulus interval) presented to Schaffer collaterals and the amplitude of the fEPSPs evoked in the CA1 layer, corresponding to the first (black circles) and the second (white circles) pulses. For each stimulus intensity, circles represent the average of the response for all the animals of each treatment group. To facilitate interpretation of the data, error bars have been omitted and the best sigmoid fit to data illustrated for each group of animals (*r* ≥ 0.985 in all cases except for the 2nd pulse of A*β* group, *r* = 0.977; *p* < 0.001). Note the increased amplitude evoked by the 2nd stimulus in the A*β-*injected animals (**B**) and how ML297 was able to restore this effect to control (vehicle) values (**C**). Right panel of (**B**,**C**) and (**D**), scatter plots and linear fit (black full lines) illustrating fEPSP amplitude values evoked by the paired-pulses in all experimental groups *vs*. control (x-axis, vehicle; y-axis, experimental group). Dashed line represents linear fit for control conditions (vehicle *vs*. vehicle) and is the same in (**B**,**C**) and **D**). Values were shifted and the slope of the linear fits was compared to control to study changes in excitability. (**E**) PPF was evoked by ≈35% of the current amount required to evoke a saturating response. Averaged fEPSP (n = 5) paired traces for each animal were collected at interstimulus intervals of 10–500 ms. Data shown are mean ± SEM amplitudes of the second fEPSP expressed as the percentage of the first [(second/first) × 100] for each of the 6 interstimulus intervals used in this test (PP ratio) for each experimental group. (**F**) Representative averaged records of fEPSPs (5 responses) recorded in the CA1 area following paired stimulation (40 ms interstimulus interval) of the ipsilateral Schaffer collaterals at 2 different intensities (1, 0.16 mA; 2, 0.34 mA; intensities 1 and 2 are indicated in B–D).

To ascertain whether activation of GirK channels prevents the potential impairments produced by A*β*, we analyzed the functional capabilities of the CA3–CA1 synapse in alert behaving mice^26^ by generating input/output (I/O) curves and testing facilitation evoked by the presentation of a pair of pulses to Schaffer collaterals (Fig. 2). *I*.*c*.*v*. injections of A*β* were performed to generate a non-transgenic mouse model of acute A*β* pathology in the dorsal hippocampus. Vehicle, ML297 (selective activator of GIRK1-containing channels), or a combination of A*β* + ML297 were also *i.c.v*. injected.

The electrical stimulation of Schaffer collaterals in behaving mice evokes a large negative wave in the CA1 pyramidal cells, with a latency of 3.5–4 ms^27^. To study synaptic transmission, we recorded changes in amplitude of field excitatory postsynaptic potentials (fEPSPs) evoked in the pyramidal CA1 area by paired-pulse (40 ms interval) stimulation in vehicle and drug-injected mice (Fig. 2F). As shown in Fig. 2A for controls (vehicle, n = 12), the amplitude of fEPSPs evoked in the CA1 area by the first pulse (black circles) increased steadily with current strength (range 0.02–0.4 mA in steps of 0.02 mA). fEPSP amplitudes evoked by the second pulse (white circles) also increased with current intensity and were larger than those evoked by the first pulse (Fig. 2A; F_(19,418)_ = 67.7, *p* < 0.001). A*β*-injected mice presented I/O curves different from controls (Fig. 2B; n = 6), showing that A*β* induced a significant increase in the excitability of the CA3–CA1 synapse when compared with vehicle-injected mice [F_(7,112)_ = 9.4; *p* = 0.002]. Firstly, the fEPSP evoked by the first pulse was smaller than the fEPSP evoked by the control stimulus, with significant differences in the range of intensities 0.02–0.4 mA [F_(19,302)_ = 2.4; *p* < 0.05; Fig. 2B, left panel]. The scatter plot in Fig. 2B (right panel) compares fEPSP amplitudes of the first pulse collected from mice treated with vehicle during the I/O study (on the x-axis) with the corresponding values after A*β* injection (y-axis). fEPSP amplitude values for A*β* with respect to control were shifted and presented a linear slope < 1 (*b* = 0.88), in accordance with an increase in the excitability of the CA3–CA1 synapse due to A*β* treatment^27^. fEPSP amplitudes evoked by the second pulse in A*β*-injected animals were significantly higher (*p* < 0.001) than those evoked in vehicle-injected mice (Fig. 2B, left panel), also in accordance with the increased excitability. Interestingly, results from the group treated with Aβ + ML297 showed that those effects of A*β* on the I/O curve were prevented by GirK channel activation with ML297 (Fig. 2C; n = 13). In A*β* + ML297, the evolution of the first and the second fEPSP evoked by the same pair of pulses at the same range of intensities (0.02–0.4 mA) was not significantly different to that of those evoked in vehicle-injected mice (*p* ≥ 0.05 for both stimuli; Fig. 2C, left panel). The scatter plot shows that, compared with control, values for the first fEPSP were shifted to a linear slope closer to 1 (*b* = 0.94; Fig. 2C, right panel), indicating a normalized excitability of the CA3–CA1 synapse similar to that in vehicle-treated mice. In contrast, GirK channel activation by ML297 alone did not produce significant changes in the I/O curves with respect to control animals (n = 6), although values for the first fEPSP with respect to vehicle were shifted to a linear slope > 1 (*b* = 1.18; Fig. 2D, right panel), suggesting a hypoexcitability compatible with an excess of inhibition in the CA3–CA1 synapse. Taken together, these results indicate that activation of GirK channels resets the excitability levels increased by A*β* in the CA3−CA1 pathway.

Next, to further study the functional capabilities of the CA3–CA1 synapse in our experimental groups, we checked whether a typical short-term plasticity phenomenon of this synapse, paired-pulse facilitation (PPF), was altered^28^. PPF has been associated to changes in neurotransmitter release, and therefore can be used to evaluate presynaptic function^29^. In addition to the 40 ms interstimulus interval fixed for the preparation of I/O curves, we tested mice for enhancement of synaptic transmission evoked by PPF using a wide range of interstimulus intervals (from 10 ms to 500 ms) at a fixed intensity (≈35% of the amount needed for evoking a maximum fEPSP response). As illustrated in Fig. 2E, all groups presented a significant [F_(1.4,50)_ = 17.1, Greenhouse Geiser correction*, p* < 0.001] increase of the response to the second pulse at short (40–100 ms) time intervals. No significant differences between groups were observed at any of the selected (10, 20, 40, 100, 200, and 500 ms) intervals [F_(3,25)_ = 0.22, *p* = 0.87], thus suggesting not only a normal short-term hippocampal plasticity but also that the drugs used in the present work are preferentially acting at postsynaptic locations.

### GirK channel activation restores the long-term potentiation (LTP) hindered by A*β* at the hippocampal CA3–CA1 synapse

**Figure 3:**
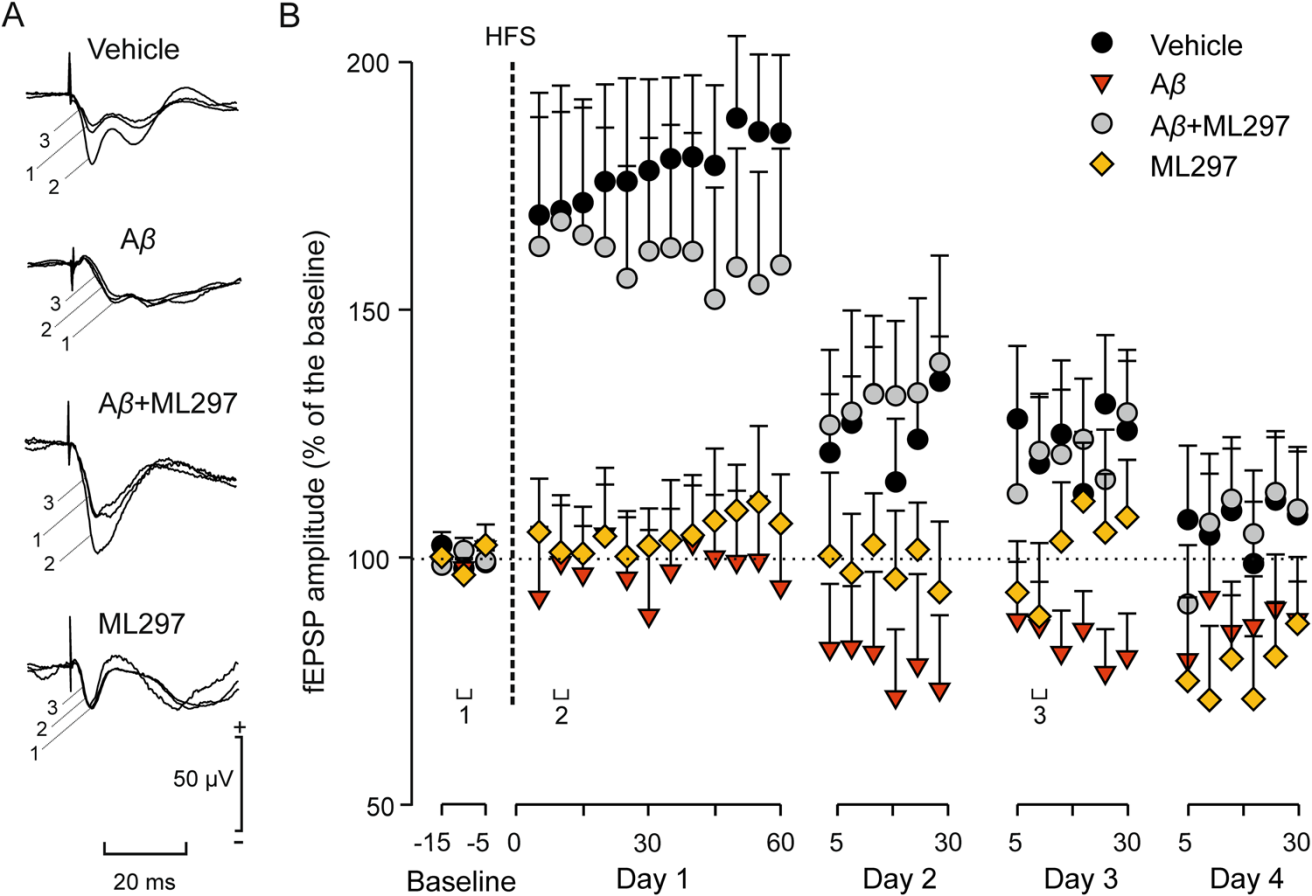
Evolution of fEPSPs evoked in the CA1 area by stimulation of Schaffer collaterals after an HFS session. For LTP induction, each animal was presented with an HFS session (see vertical dotted line) consisting of five 100 Hz, 100 ms trains of pulses at a rate of 1/s. This protocol was presented 6 times at intervals of 1 min. The 100-μs, square, biphasic pulses used to evoke LTP were applied at the same intensity used for the single pulse presented following HFS presentation. The evolution of LTP was checked using a single pulse (**A**) Representative examples (averaged 5 times) of fEPSPs evoked at the CA3–CA1 synapse by single stimulation collected following the respective injection but prior to HFS (1, baseline), 10 min after HFS (2), and 48 h after HFS (3). (**B**) Illustrated data (mean ± SEM) correspond to LTP evoked in controls, A*β*, A*β* + ML297, and ML297 groups.

To further investigate whether enhancing GirK channels activity might compensate deficits in synaptic plasticity in our non-transgenic mouse model, we induced LTP by high-frequency stimulation (HFS) of the hippocampal Schaffer collateral pathway, and compared the evolution of fEPSPs evoked at the CA3–CA1 synapse in freely moving mice (Fig. 3). Twenty-four hours after *i.c.v*. injections, animals were stimulated with single pulses for 15 min (at a rate of 3/min) at Schaffer collaterals in order to obtain a baseline for evoked fEPSPs (Fig. 3B). For LTP induction, each animal received an HFS session (dotted line, Fig. 3B), its evolution was checked during the following 60 min and, in addition, for 30 min on the following 3 days after the HFS session, presenting the same pulses at Schaffer collaterals. With this protocol, LTP was induced in vehicle-injected animals, but not in the A*β* and ML297 groups (Dunnett’s T *post hoc vs*. vehicle: A*β, p* = 0.03; ML297, *p* = 0.04). Specifically, and compared with fEPSP amplitude values recorded during the baseline, after the injection control mice presented a mean potentiation of ≈178 ± 5% (Fig. 3B, black circles) during the 60 min following HFS [F_(14, 154)_ = 12.7, *p* = 1.7 × 10^−19^]. LTP induced in control animals remained significantly larger than baseline values at least 24 h after the HFS session [F_(2.3, 88_ = 5.1 Greenhouse-Geisser correction, *p* = 0.01)]. Interestingly, mice injected with both A*β* + ML297 presented LTP values similar to those reached by the control group (Fig. 3B, white circles) after the HFS session (mean potentiation ≈161 ± 7%; Dunnett’s T *post hoc vs*. vehicle *p* = 0.83), indicating that the opening of GirK channels is able to restore this important memory-related hippocampal property when it is blocked by the presence of A*β*.

### GirK channel opening is also able to recover power spectra of the hippocampal oscillatory activity in A*β*-injected behaving mice

**Figure 4:**
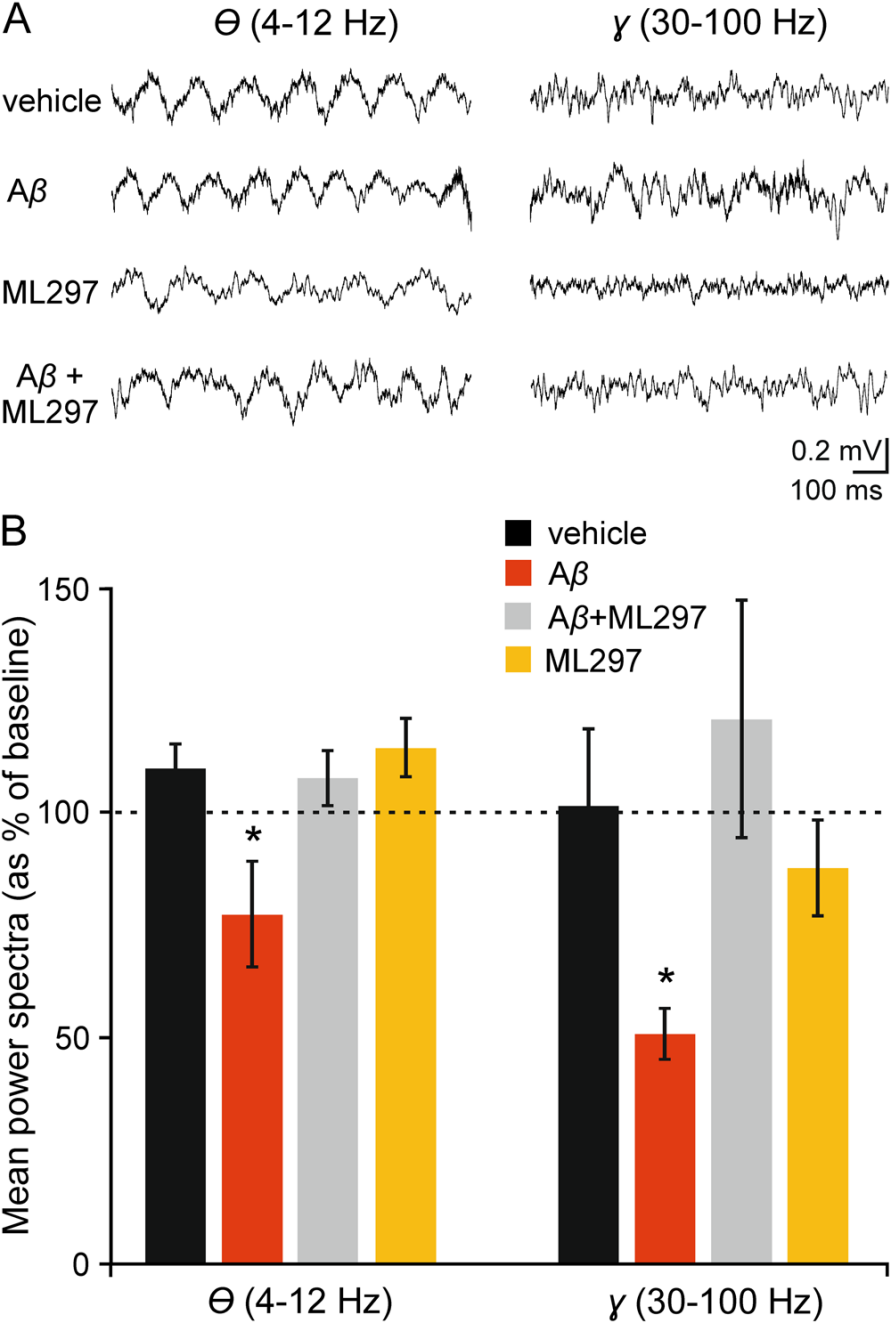
Spectral power in *theta* and *gamma* bands of EEG collected from hippocampal pyramidal CA1 areas. (**A**) LFP/EEGs recordings were carried out for 5 min, from each of which up to 3 min of recording free of unwanted artifacts was selected for spectral analysis. Baseline values were obtained before the injections were performed. (**B**) Histograms represent the spectral power of LFP activity recorded in CA1 hippocampal region after injections were performed, as percentage of baseline recordings (dashed line, 100%). Values of selected spectral bands (4–12 Hz, 30–100 Hz) for *theta* and *gamma* oscillations are shown. Note that while ML297-alone injection has no effect on the mean power values analyzed, A*β*-injected animals showed significantly decreased values for both spectral bands with respect to control (vehicle). The presence of the GirK opener ML297 was able to rescue the control power spectra values for *theta* and *gamma* rhythms when it was injected together with A*β*. *p < 0.05 *vs*. vehicle.

As hippocampal network activity is known to be altered in transgenic^4,5^ and acute^30^ AD models, we next examined in our model the role of GirK channels in the oscillatory network activities of hippocampal circuits recorded in behaving mice with chronically implanted electrodes. Field hippocampal activity was recorded to determine the power spectra of the *theta* (4–12 Hz) and *gamma* (30–100 Hz) bands, which are both known to be dramatically altered in AD models^31,32^, and was normalized as the percentage of the pre-injected values (baseline) (Fig. 4). *I.c.v*. injection of A*β* induced a significant decrease of the power for *theta* and *gamma* bands when compared with vehicle-injected mice (*post hoc vs*. vehicle: *p* < 0.05 for both *theta* and *gamma*). No changes were observed in ML297-injected animals in either of the bands. Conversely, in the A*β* + ML297 group, the power spectra for both bands was recovered to control values (*p* ≥ 0.05 for both bands), suggesting that the enhancement of GirK activity is enough to compensate A*β*-induced deficits in these hippocampal network activities crucial for accurate learning and memory processing.

### Increasing GirK activity restores long-term object recognition memory previously impaired by hippocampal A*β* injection

**Figure 5:**
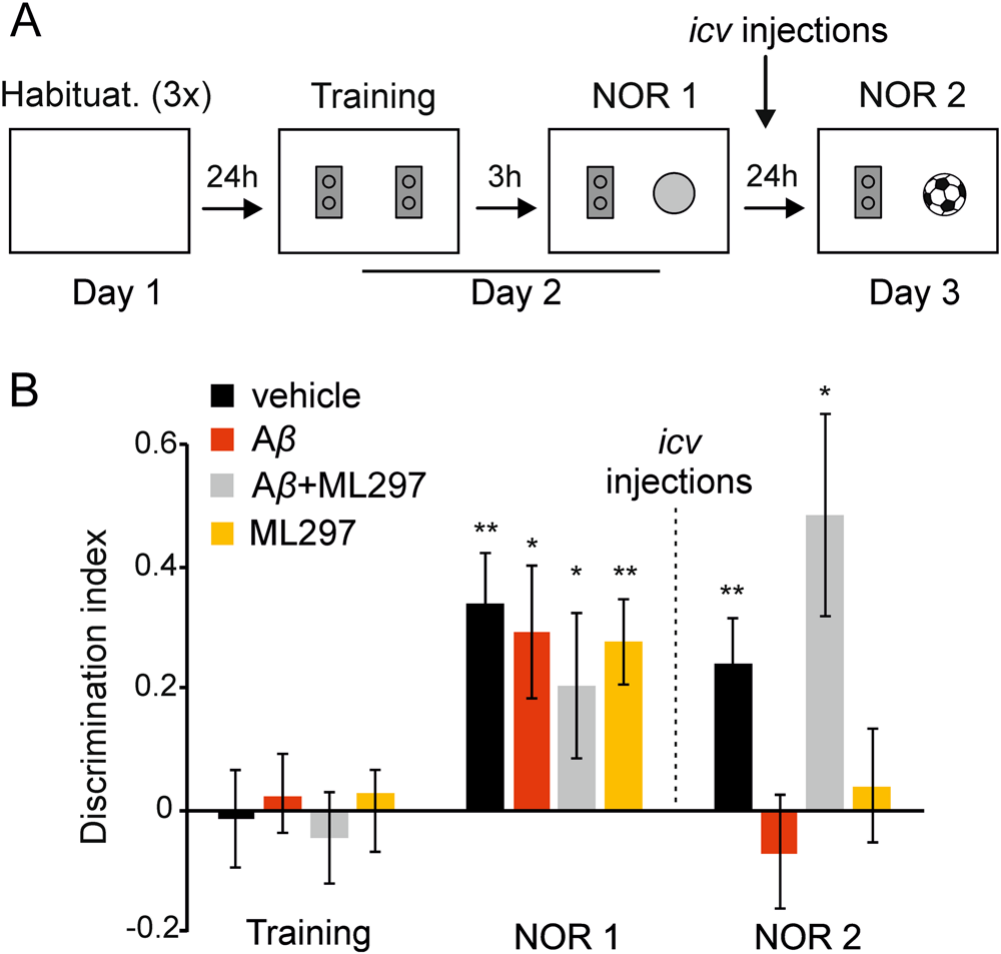
Novel object recognition. (**A**) NOR protocol consisted of three 5-minute habituation sessions to the empty arena (1.5 h of interval between sessions) on day 1. On day 2, two identical objects (yellow Lego pieces) were placed in the center of the arena and animals were allowed to explore for 10 min (training session). After 3 h, one object was replaced by a new one (pink cylinder) for the first test session (NOR1). On day 3, animals were *i.c.v.-*injected with A*β*, ML297, or both. Mice injected with vehicle were used as a control group. A second test (NOR2) was conducted 1 h after injections, with the familiar object and a new novel one (mini football ball). (**B**) Histograms represent the discrimination index (DI) for each experimental group during training, NOR1 and NOR2 tests. DI is defined as the difference in exploration time (T) between the two objects (O), divided by the total time spent exploring both objects: DI = (TO1−TO2)/(TO1+TO2). TO1 and TO2, time exploring object 1 and object 2, respectively. DI = 0, no discrimination. *I.c.v. i*njections are indicated by a dashed line between NOR1 and NOR2. *p < 0.05; **p < 0.01; ***p < 0.001 *vs*. same group in the training session. Habituat., habituation.

Having established that, in the hippocampus, activation of GirK channels is able to restore deficits induced by A*β* at the synaptic, circuit, and network levels, we wondered whether these impairments would have a behavioral correlation and, if so, whether increasing GirK activity might have beneficial effects on impaired learning and memory in our acute AD mouse model. Given that Object Recognition (OR) memory formation is one of the early traits of cognitive decline observed in AD patients^33^, and is totally reliant on CA3–CA1 synaptic functionality in behaving mice^34^, we used the Novel OR (NOR) test to answer the above question. The task relies upon the tendency of rodents to attend to a novel object more than to a familiar one. First, animals were habituated to the NOR arena in three successive habituation sessions (Fig. 5A). Locomotor activity significantly decreased across the sessions as evidenced by the reduction in the percentage of crossings [F_(1.62, 73)_ = 223.3 Greenhouse-Geisser correction, *p* < 0.001] and rearings [F_(1.64, 73)_ = 69.2 Greenhouse-Geisser correction, *p* < 0.001] (data not illustrated). Afterwards, during the training session, all animals spent a similar amount of time exploring each object (50% of time on each), yielding a discrimination index (DI) of approximately 0 (DI = 0 accounts for no discrimination or preference between objects; Fig. 5B; *p* ≥ 0.05 for all experimental groups). In contrast, during the test session 1 (NOR1), performed 3 h after training, all animals showed a strong preference for exploring the novel object (Fig. 5B, DI = 0.34 ± 0.08, *t(7)* = 4.1, *p* = 0.004 for vehicle; DI = 0.29 ± 0.1, *t(6)* = 2.7, *p* = 0.03 for A*β*; DI = 0.29 ± 0.09, *t(6)* = 2.9, *p* = 0.02 for A*β* + ML297; DI = 0.3 ± 0.07, *t(8)* = 3.9, *p* = 0.004 for ML297) with no differences between groups [F_(3, 30)_ = 0.11, *p* = 0.95), indicating that short-term memory (STM) was intact in these animals what very likely meant that synaptic plasticity processes (i.e. LTP in the hippocampal CA3–CA1 pathway) were functional *in vivo* in animals that would perform subsequent NOR2. On the next day after NOR1, mice were injected *i.c.v*. with either vehicle, A*β*, ML297, or a combination of A*β* + ML297. One hour after drug injections long-term memory (LTM) was assessed in these animals by an additional NOR trial (NOR2). As OR memory formation is totally dependent on CA3–CA1 synaptic functionality, an experimental procedure such as A*β* injection capable of disturbing hippocampal patterns of synaptic strength^35,36^, would be enough to prevent memory^34^. As expected, vehicle injections did not change object recognition (DI = 0.24 ± 0.1, *t(7)* = 3.1, *p* = 0.01) while A*β-*injected mice explored both objects equally (DI = −0.07 ± 0.1, *t(6)* = −0.8, *p* = 0.45), showing an impaired NOR LTM formation. This memory impairment was also found in ML297-treated animals (DI = 0.03 ± 0.1, *t(8)* = 0.4, *p* = 0.7). However, injection of both A*β* + ML297 significantly increased the exploration of the novel object (DI = 0.5 ± 0.17, *t(6)* = 2.9, *p* = 0.032) to a level similar to that of vehicle animals (*post hoc vs*. vehicle, *p* = 0.64). No significant changes between experimental groups (*p* ≥ 0.05, data not shown) were observed in the total amount of time spent exploring the objects in any of the three sessions (Training, NOR1, or NOR2).

In summary, our results suggest that hippocampal alterations found at the synaptic, circuit, and network levels are also translated into behavior deficits, and increasing GirK activity shows beneficial effects on impaired learning and memory in our AD model.

## Discussion

Increasing evidence shows that, at preclinical stages of AD, subtle changes in the excitation/inhibition balance triggers hyperactivity as an early neuronal dysfunction^5^, underlying upstream consequences on synaptic, circuit, network, and behavioral levels^4,30,37,38^. Although the mechanism underlying the increased excitability remains unclear, current therapies targeted at increasing the inhibition are clearly showing promising results in animals^7,8,39,40^, and even in humans^6^.

Neuronal hyperexcitability can be generated by A*β*-induced impairment in the maintenance of resting membrane potential through mechanisms involving alteration in receptors −purinergic^41^ or muscarinic^42^− and channels such as A-type potassium^43,44^ or GirK^20^. Soluble A*β*_1-42_ peptide is a key pathological specie in AD widely used due to its potential contribution to both pathogenesis and disease progression^45^. In hippocampal brain slices acute application of A*β*_1-42_ is known to increase excitability^35,46^, impair LTP^47^, and decrease *theta*^31^ and *gamma*^48^ band powers. *In vivo*, besides hippocampal-dependent behavioral deficits^49^ and LTP impairment^50^, the application of A*β*_1-42_ induces a degradation in *theta*^49^ and *gamma*^51^ oscillatory activities. Our data show that A*β*_1-42_ generates an early local AD-like pathology in mice, characterized by an increased excitability in the dorsal hippocampus synapse together with a disruption of processes necessary for learning and memory, such as LTP and oscillatory network activity in the *theta* and *gamma* ranges. We also found that by activating GirK channels, hippocampal A*β*-induced disruptions are counteracted.

GirK channels play an important role in the regulation of neuronal excitability. In our experiments, synaptic responses were recorded from dendrites of dorsal CA1 pyramidal neuron region (Fig. 1)^26^ where GIRK1/GIRK2 channels are mostly located in adults^14,52,53^. At this location GirK channels are crucial for the maintenance of the resting membrane potential due to a continuous and sustained activity^10,12^, therefore their activation could reduce the propagation of synaptic excitation from the distal dendrites to the pyramidal cell soma^52^. The latter would be beneficial for re-establishing a balance in a system altered by A*β*^4,37^, as we showed when injection of A*β* is followed by ML297, a specific agonist for GIRK1-containing heterotetramers^54,55^. Available evidence suggests that enhanced GirK-dependent signaling is detrimental to cognition and normal synaptic plasticity by an excess of inhibition^11,17,56^. It has been proposed that the larger resting GirK conductance in dorsal CA1 neurons underlies the higher threshold for LTP induction^12^. In the ML297-alone injected mice, the hyperactivity of that strong component of the inhibitory conductances may underlie this inhibition of LTP. However, in an altered system such as AD, where the excitation/inhibition ratio is increased, the restoring effects of activating GirK channels at the synaptic level could induce the re-establishment of the membrane potential value impaired by A*β*^4,18^. This rescue counteracts the hyperexcitability of the hippocampus as shown by the I/O curves.

On the other hand, we did not find changes in the PPF ratio. This result indicates not only that this typical presynaptic short-term plastic property of the CA3–CA1 synapse is not significantly altered by A*β* or the modulation of GirK channels, but also that the effect of the drugs used is produced mainly at postsynaptic sites, thus acting on pyramidal neurons^57,58^. Indeed, although GirK channels are also found in CA1 interneurons^53,59^ which are target of A*β*^25^, the *net effect* we obtained is more compatible with effects on pyramidal cells.

For memory formation, neuronal synchronization is a capital point. In multiple brain regions, including the hippocampus, synchronization of action potentials provides a link between oscillatory activity and cellular plasticity mechanisms^60^ underlying the spike time-dependent plasticity phenomena^61^. For example, during slow oscillations such as *theta* activity, LTP is induced by pairing EPSPs with depolarizing peaks of oscillations, whereas LTP is reset when the stimulus is paired with hyperpolarizing phases^62^. These properties can also be observed during high-frequency oscillatory activity such as *gamma* range, where small shifts in synchrony induce modifications in synaptic plasticity^63^. These oscillations interact with each other to generate the so-named *theta*–*gamma* neural code, involved in memory processes^64^. These findings suggest that *theta* and *gamma* oscillations in the hippocampus act as a windowing mechanism for LTP^65^ and might explain the deficits induced by A*β* in LTP and the NOR test in our AD model. In fact, it has been proposed that *theta*–*gamma* uncoupling might represent an early electrophysiological signature of hippocampal network dysfunction in AD^66,67^. But how could the enhanced GirK signaling oppose the effects of A*β* on network properties? GABAergic neurons modulate pyramidal cell activities and support *theta* oscillations^68^. It has been shown that A*β* progressively impairs behavioral performance and associated hippocampal *theta* power^49^ by a specific reduction in the firing of rhythmic bursting GABAergic septohippocampal neurons^69^. The enhancing of GirK activity in pyramidal neurons could compensate this deficit and explain the re-establishment of *theta* power. We also found GirK activation to increase *gamma* power reduced by A*β*, which is beneficial in AD models^8^. It has been shown that *gamma* oscillations might be impaired by an increased excitability in CA1 pyramidal cells *via* a decreased expression of GIRK2 channel protein expression^70^. Similar results have previously been reported in CA3 pyramidal cells where GirK channels, acting as effector of serotonin receptors, help the brain to maintain or re-establish normal *gamma* oscillation levels^71^. Hence, strategies to increase *gamma* oscillation through GirK activity enhancement should be considered in AD models. This idea is strongly supported by recent findings regarding GirK functioning. The massive influx of Na^+^ ions due to high-frequency bursts of excitation, such as *gamma* or wave-ripple activity, both critical for memory formation and consolidation in the hippocampus^60^, induces an amplified neuronal inhibition mediated by GirK channels^72^. This mechanism would be compatible with our results, as we hypothesized that in an A*β*-induced *gamma* disruption scenario, increasing GirK-signaling with ML297 would mimic the physiological boosting of inhibitory activity needed to counteract the hyperactive neurons, helping memory processing.

From the experiments presented here, it appears that memory processing during NOR tests was disabled in the presence of A*β* or by GirK channel activation alone. In accordance with these results, the enhancement of GirK signaling in naïve animals has been shown to be detrimental to cognition^9^, while altered recognition of familiar and novel objects has already been found in AD patients^33^. NOR memory formation relies on proper CA3−CA1 synaptic functionality^34^, and the lack of LTP in this region is correlated with an impairment of non-spatial hippocampal memory formation^34,73^. In our experiments LTP impairment was induced by A*β*. This type of synaptic plasticity was also hindered in ML297-alone injected mice most likely due to large hyperpolarization of dorsal CA1 pyramidal neurons induced by GirK opening^12^, as it has also been shown for GABA_B_ agonists^74^. However, when LTP is impaired by A*β*-induced hyperexcitation, the repolarizing effect of GirK-signaling enhancement on CA1 pyramidal neurons would allow reaching control values in NOR scores. These data suggest that although we previously found A*β* to alter GirK channels at the molecular^19^ and synaptic^20^ levels in the hippocampus, its effects *in vivo* on hippocampal excitability cannot only be attributed to a modulation of such channels.

In summary, the results presented here show that GirK channels play a role in linking hyperexcitability, impaired synaptic plasticity, and cognitive deficits in an acute model of AD. One may suspect that this would also happen where neuronal hyperactivity is an early dysfunction, as already reported in genetic models due to non-deposited A*β* forms^4,5^ or in AD patients at initial phases^6,23,38^. In this sense, GirK channels provide an important regulatory function in hippocampal principal neurons, particularly in the temporoammonic inputs, that must be explored as a promising approach for the development of efficient treatment of AD in preclinical stages.

## Methods

### Subjects

Experiments were performed on 90 C57BL/6 male adult mice (3–5 months old; 28–35 g) obtained from an official supplier (Janvier, France). Before surgery, animals were housed in separate cages (n = 5 per cage). The mice were kept on a 12 h light/dark cycle with constant ambient temperature (21 ± 1 °C) and humidity (50 ± 7%). Food and water were available *ad libitum*. We considered successful experimental animals only those that reached all the behavioral criteria and had appropriate electrode placements, as checked histologically. Electrical recordings selected for analysis had to display clear fPSP components in the absence of any sign of epileptiform activity (stimulus-evoked after-discharges, and/or ictal or post-ictal activity), and extracellular recordings [i.e., fPSPs and/or local field potentials (LFPs)] that did not deteriorate over time. The number of successful animals used per experimental group was n = 6−13, depending on the experiment.

### Ethics

All experiments were performed in accordance with European Union guidelines (2010/63/EU) and with Spanish regulations for the use of laboratory animals in chronic experiments (RD 53/2013 on the care of experimental animals: BOE 08/02/2013), and approved by the local Ethics Committees of the Universities of Castilla-La Mancha and Pablo de Olavide.

### Surgery for chronic recordings in behaving animals

C57BL/6 male mice were anesthetized with 4% chloral hydrate. A total of 90 animals were implanted with bipolar stimulating electrodes aimed at the right Schaffer collateral-commissural pathway of the dorsal hippocampus (Fig. 1A,B; 2 mm lateral and 1.5 mm posterior to bregma; depth from brain surface, 1.0–1.5 mm^75^, and with a recording electrode aimed at the ipsilateral *stratum radiatum* underneath the CA1 area (1.2 mm lateral and 2.2 mm posterior to bregma; depth from brain surface, 1.0–1.5 mm). These electrodes were made from 50-μm, Teflon-coated tungsten wire (Advent Research Materials, UK). The final position of the hippocampal electrodes was determined using as a guide the field potential depth profile evoked responses presented at the Schaffer collateral pathway (Fig. 1C)^26^.

For the *i*.*c*.*v*. administration of drugs included in this study, the selected animals were also implanted chronically with a blunted, stainless steel, 26-G guide cannula (Plastic One, Roanoke, VA, USA) in the ventricle (0.5 mm posterior to bregma, 1.0 mm lateral to midline, and 1.8 mm below the brain surface^75^), contralateral to the hippocampal stimulating and recording electrodes (Fig. 1D). Injections were carried out with a 33-G internal cannula, 0.5 mm longer than the implanted guide cannula and inserted inside it. Injections in the freely moving mice were performed with the help of a motorized Hamilton syringe at a rate of 0.5 μL/min. *I.c.v*. injections were performed after baseline LFP recordings and 24 h before I/O, PPF and LTP protocols, and 1 h before LTM testing during NOR task (see details below).

A bare silver wire (0.1 mm) was affixed to the skull as a ground. The four electrodes and the ground were connected to a 6-pin socket and the socket was fixed to the skull with the help of two small screws and dental cement^26^. Mice were allowed a week for recovery before the experimental sessions. Handling was performed routinely to minimize stress to the mice during experimental manipulation.

### Local field potential recordings, input/output curves, and paired-pulse facilitation

The LFP activity and fEPSPs were recorded from alert behaving mice with Grass P511 differential amplifiers through a high-impedance probe (2 × 10^12^ Ω, 10 pF). LFPs were recorded from the hippocampal CA1 area in the absence of any electrical stimulation of Schaffer collaterals, with the behaving animal placed in a small (5 × 5 × 5 cm) box before (baseline values) and after *i.c.v*. injections. Recordings were performed for 5 min from which up to 3 min of recording, free of unwanted artifacts, was selected for spectral analysis. We selected the following frequency bands: *theta*, 4–12 Hz^76,77^ and *gamma*, 30–100 Hz^78^. The power spectrum of the hippocampal activity was computed with Spike2 software, using the fast Fourier transform with a Hanning window and expressed as percentage of power spectrum change with respect to mean pre-injected values.

For fEPSPs, electrical stimuli presented to Schaffer collaterals consisted of 100 *μ*s, square, biphasic pulses presented alone, paired, or in trains. For the construction of the I/O curves, stimulus intensities ranged from 0.02 mA to 0.4 mA and were elicited at 40 ms of interstimulus interval. For the PPF protocol, the stimulus intensity was set well below the threshold for evoking a population spike, ∼35% of the intensity necessary for evoking a maximum fEPSP response^26^.

### Long term potentiation

For LTP induction in behaving mice, the stimulus intensity was also set at ∼35% of its asymptotic value. An additional criterion for selecting stimulus intensity for LTP induction was that a second stimulus, presented 40 ms after a conditioning pulse evoked a larger (≥150%) synaptic field potential than the first^79^. Baseline (BL) values for the amplitude of fEPSPs evoked at the CA3–CA1 synapse were collected 15 min prior to LTP induction using single 100 μs, square, biphasic pulses elicited at 0.05 Hz. For LTP induction, animals were presented with an HFS session consisting of five 100 Hz, 100 ms trains of pulses at a rate of 1/s repeated 6 times, at intervals of 1 min—that is, a total of 300 pulses were presented during the HFS session^26^. To avoid evoking large population spikes and/or the appearance of cortical seizures, the stimulus intensity during HFS was set at the same intensity as that used for generating BL recordings. After the HFS session, exactly the same single-stimulus parameters as for BL recordings were presented for the following 60 min. On following days, the BL parameters were used for recording sessions lasting for 30 min. All HFS data were normalized using BL fEPSP values collected on the first day as 100%; this way, we could evaluate early and late LTP.

### Novel object recognition protocol

NOR experiments to test long-term memory (LTM) were conducted in a uniformly illuminated open-field arena (30 × 25 × 20 cm) built of polyvinyl chloride plastic, plywood, and transparent acrylic as previously described^34^. Stimulus objects were made of plastic. There were several copies of each object, which were used interchangeably. All objects had been previously tested (validated) with a separate group of mice to ensure no preference of the animals for any particular object due to its shape, color, size, etc. For object validation^34^, it was verified that the exploration time of a pair of objects was equal when they were presented for the first time to a group of animals (minimum n = 5). Thus, objects that presented preference over a previously validated one, were eliminated from the study. This validation test was performed in experimental conditions identical to the rest of the test. The relative position of the two stimulus objects was counterbalanced and randomly permuted for each experimental animal. All objects were static (always attached to the floor of the open-field arena). The open-field arena and the stimulus objects were cleaned thoroughly between trials to ensure the absence of olfactory cues. Exploration was defined as sniffing or touching the stimulus object with the nose and/or forepaws, or pointing the nose towards the object from a distance <1 cm. Sitting on or going around the objects was not considered exploratory behavior. A video camera was positioned over the arena and the behavior of the mice was recorded using a video tracking and analysis system. The experiments were performed by an observer blind to the treatment condition of the animals. During all behavioral sessions, lights were kept dim (30–40 lx).

The NOR task consisted of three 5-minute habituation trials performed on the first day of experiment followed by three 10-minute trials distributed over a two-day period (i.e. performed on the second and third days of experiment). In each habituation session, mice were allowed to explore the arena freely for 5 min in the absence of any other behaviorally relevant stimulus. Habituation sessions were performed every 90 min for a single mouse on the same day (day1). In order to test that animals were able to learn the NOR task and therefore, to consolidate and retrieve OR memory properly, in the second day of experiment, OR training occurred: mice were placed in the open-field arena containing two identical objects (two Lego pieces) and left to explore them freely for 10 min (acquisition/training session). A 10-minute test session was performed 3 h later (NOR1, day 2) for evaluation of short-term memory (STM) retention^34^. For this purpose, one of the objects used at training was randomly substituted by a novel one (cylinder) and exploratory behavior of the mice toward familiar and novel objects was quantified. Only data from animals that learned successfully (≈92% of subjects)—i.e., explored the new object for significantly longer than the familiar one—were analyzed and included in additional tests. On the third day of experiment an additional NOR trial (NOR2) was performed to test LTM retention. Retrieval of a consolidated memory in the presence of novelty is called reconsolidation, a labile phase of the consolidated memory that requires stabilization to persist. LTP has been correlated with all distinct phases of NOR memory formation and retrieval, including reconsolidation^34^. Therefore, impairment of LTM in our experimental design would very likely imply LTP disruption. For NOR2 test, animals were allowed to explore the open field for 10 min and the novel object used at NOR1 was again replaced by a novel one (mini football ball, NOR2). Exploratory behavior of the mice toward novel and familiar object was measured. To analyze the impact of A*β* or GirK channel activation on LTM retention, *i.c.v*. injections of either vehicle, A*β*, ML297, or A*β* + ML297 were performed as described above 1 h before the NOR2 test. Learning was evaluated by quantification of the relative exploration time of each object and determination of the Discrimination Index (DI), defined as the difference in exploration time between the two objects (TO1-TO2; TO1 and TO2 indicate time exploring object 1 and object 2 respectively), divided by the total time spent exploring both objects (TO1 + TO2). DI = (TO1 − TO2)/(TO1 + TO2). If we consider O1 to be the Novel Object, a DI close to 1 will indicate a higher preference of exploration for the said object.

### Drugs

All chemicals used in this study were purchased from Abcam (Cambridge, UK) and dissolved in PBS with the help of a shaker and/or sonicator. To model focal A*β* pathology in the dorsal hippocampus *in vivo*, we set up a non-transgenic mouse model^80–82^ that resembles initial preclinical stages of the disease and enables evaluating the key role of early amyloid forms in AD. A*β*_1-42_ was dissolved in vehicle and incubated 1 h at room temperature before injection to form soluble oligomers and not fibrils according to the protocol described by Jan and collaborators^83^. For *i.c.v*. injection, 3 μg of A*β* were dissolved in 3 μL of vehicle and injected through the cannula using a Hamilton syringe at a rate of 0.5 μL/min. Doses of the selective activator of GIRK1– containing channels, ML297 (1.5 mM), were determined following preliminary tests and based on previous reports^84,85^, and dissolved in 3 μL for injection. The A*β* + ML297 group was firstly injected with A*β* and 15 min later with ML297.

### Histology and immunohistochemistry

To verify the proper location of implanted electrodes and cannulas, at the end of the experiments mice were deeply anesthetized (4% chloral hydrate, 0.5 mL/mouse) and perfused transcardially with saline followed by 4% paraformaldehyde in phosphate-buffered saline (PBS, 0.1 M, pH 7.4). Their brains were removed and cryoprotected with 30% sucrose in PB. Coronal sections (40 *µ*m) were obtained with a sliding freezing microtome (Microm HM 450, Walldorf, Germany) and stored at −20 °C in 50% glycerol and 50% ethylene glycol in PB until used. Selected sections including the implanted sites were mounted on gelatinized glass slides and stained using the Nissl technique with 0.25% Thionine to determine the location of stimulating and recording electrodes and/or the implanted cannula (Fig. 1A,D). For immunohistochemistry (Fig. 1B), free-floating sections were treated for 45 min with 10% normal donkey serum (Sigma Aldrich, Poole, UK) in Tris-buffered saline (TBS) containing 0.1% Triton X-100, and then incubated overnight at room temperature with polyclonal rabbit anti-GirK1 subunit (1:200; Alomone Labs, Jerusalem, Israel) primary antibody. The following day, sections were washed with TBS with 0.1% Triton X-100 (3 × 10 min) and incubated for 2 h at room temperature with 1:150 dilutions of FITC-conjugated donkey-anti-rabbit (Jackson Immuno Research, West Grove, US). Sections were then washed with TBS (3 × 10 min) and incubated in 0.01% DAPI (Santa Cruz Biotechnology, Santa Cruz, US) in TBS for 5 min. Finally, sections were washed with TBS (3 × 10 min), mounted on gelatinized glass slides, dehydrated and coverslipped using a fluorescence mounting medium (Dako mounting medium, Agilent, Santa Clara, US). Images were acquired by confocal microscopy using a laser scanning microscope (LSM 800, Carl Zeiss, Jena, Germany).

### Data collection and analysis

Recordings were stored digitally on a computer through an analog/digital converter (CED 1401 Plus). Data were analyzed off-line for quantification of LFPs and fPSPs, using the Spike2 (CED) program and the video capture system. Since synaptic responses did not shown contamination by population spikes, the amplitude (i.e., the peak-to-peak value in mV during the rise-time period) of 5 successively evoked fPSPs was computed and stored for later analysis^86^. These computed results were processed for statistical analysis using the SigmaPlot 11.0 package (SigmaPlot, CA, USA). Figures were prepared using CorelDraw × 7 Software.

### Statistical analysis

Unless otherwise indicated, data are represented as the mean ± SEM. All calculations were performed using SPSS version 20 software (SPSS Inc., Chicago, IL). When the distribution of the variables was normal, acquired data were analyzed with the two-tailed Student’s *t* test or the one-way or two-way ANOVA, with *time* and *treatment* as within- and between-subjects factors respectively (except in I/O experiments in which intensity was the repeated measure), and with a contrast analysis for a further study of significant differences. For repeated measures two-way ANOVA, Greenhouse Geiser correction was used and indicated in the text when sphericity was not assumed. If the Levene test for normal distribution was significant then data were normalized by logarithmic (ln) transformation. This was the case for I/O and PPF data as well for LFP/EEG data for the *gamma* band. Statistical significance was set at *p* < 0.05.

### Data availability

The datasets generated during and/or analyzed during the current study are available from the corresponding author on reasonable request.
