# Publisher Correction: Activation of G-protein-gated inwardly rectifying potassium (Kir3/GirK) channels rescues hippocampal functions in a mouse model of early amyloid*-β* pathology

**DOI:** 10.1038/s41598-020-58226-w

**Published:** 2020-01-29

**Authors:** Irene Sánchez-Rodríguez, Sara Temprano-Carazo, Alberto Nájera, Souhail Djebari, Javier Yajeya, Agnès Gruart, José M. Delgado-García, Lydia Jiménez-Díaz, Juan D. Navarro-López

**Affiliations:** 1 University of Castilla-La Mancha, NeuroPhysiology & Behavior Laboratory, Centro Regional de Investigaciones Biomédicas, School of Medicine of Ciudad Real, Ciudad Real, Spain; 2 University of Salamanca, Instituto de Neurociencias de Castilla y León, Salamanca, Spain; 3 Pablo de Olavide University, Division of Neurosciences, Seville, Spain

Correction to: *Scientific Reports* 10.1038/s41598-017-15306-8, published online 07 November 2017

The original version of this Article contained an error in Affiliation 1, which was incorrectly given as ‘University of Castilla-La Mancha, NeuroPhysiology & Behavior Laboratory, Centro Regional de Investigaciones Biomédicas, Albacete, Spain’. The correct affiliation is listed below:

University of Castilla-La Mancha, NeuroPhysiology & Behavior Laboratory, Centro Regional de Investigaciones Biomédicas, School of Medicine of Ciudad Real, Ciudad Real, Spain

This error has now been corrected in the HTML and PDF versions of the Article.

